# Rural Housing and Rural Sickness

**Published:** 1924-04

**Authors:** 


### RURAL HOUSING AND RURAL SICKNESS.

AMID the din of talk about housing very little is heard of the rural side of the problem. The towns have taken the floor, they occupy so much space, and make so much noise that the still small voice of the villages can hardly be distinguished. Housing Bills, including the egregious measure now before a Standing Committee of the House of Commons, are based upon urban needs and either misunderstand or ignore the rural problem. How acute that problem is and how gravely it affects the health of village dwellers is significantly indicated by the answers to a *questionnaire* that has been sent by the Leicestershire Health Insurance Committee to the panel doctors in the county. From the replies to the Committee's questions it appears that the Leicestershire villages suffer from "most unsatisfactory and inadequate" housing conditions. The figures relate to 1922, and show that, in consequence of the prevalence of damp, and insanitary houses and their inadequate accommodation, diseases of the respiratory organs are the most prevalent troubles. Next to them comes heart disease, and the third place is occupied by cancer, which accounted for $10\frac{1}{2}$ per cent. of the year's deaths.

Were the respiratory diseases not lumped together cancer would come second in the melancholy list, and it would appear to be only a matter of time before it stands at the head of it. In 1900 the deaths from cancer in Leicestershire were one in every thirty-one; in 1922 they were one in ten and a-half, as compared with one in just under thirteen from heart disease. Thus cancer is now three times as fatal in Leicestershire as it was less than a quarter of a century ago. To what extent overcrowding and lack of sanitation in the broad sense of the word are responsible for these terrible figures no man may say, our knowledge of the causation of cancer being still elementary; but we know enough of the subject to be entitled to declare that these things must necessarily be serious factors. The doctors appear to have been asked if there are any "cancer houses" in their districts, and one of them answers, "Not cancer houses, but cancer villages." That is to say, there are certain communities so disadvantageously situated as regards elevation, soil, humidity, or housing, or some one of these or even all of them together, that their inhabitants are predisposed to the attacks of this most mysterious and insidious disease.

Cancer by no means stands alone in a sinister relation to housing. The general view of the panel doctors in Leicestershire, as in many other parts of the country, is that the conditions of domiciliary treatment of tuberculosis are "most unsatisfactory" and go far to undo the beneficial results of sanatorium treatment. Tuberculous patients return to overcrowded and otherwise insanitary houses, where they live amid conditions which effectually preclude complete cure. Shelters of one kind or another for these patients are a mere palliative—what is needed is houses in which men and women may lead healthy lives with a reasonable probability of avoiding deadly disease. "Whole blocks of disease," Sir Leslie Mackenzie has just been saying at Glasgow, "need not exist if we choose to prevent them," and one of the most certain ways of preventing them is to see that our people live in houses which are not traps for them. It is a tremendous task full of difficult problems, and we shall not solve those problems without heavy sacrifices. But it is better to sacrifice money and even, if need be, to banish political economy to Jupiter and Saturn than to go on recklessly sacrificing life and health. If we apply ourselves steadily and sanely to the problem of village—as of urban—housing there will be little need for such banishment. The first necessity is to keep well before our minds the fact that the improvement of housing is, in its way, quite as serious a matter in the villages as in the towns.

